# VIS-NIR Diffuse Reflectance Spectroscopy System with Self-Calibrating Fiber-Optic Probe: Study of Perturbation Resistance

**DOI:** 10.3390/diagnostics13030457

**Published:** 2023-01-26

**Authors:** Valeriya Perekatova, Alexey Kostyuk, Mikhail Kirillin, Ekaterina Sergeeva, Daria Kurakina, Olga Shemagina, Anna Orlova, Aleksandr Khilov, Ilya Turchin

**Affiliations:** Institute of Applied Physics RAS, 603950 Nizhny Novgorod, Russia

**Keywords:** diffuse reflectance spectroscopy, diffuse optical spectroscopy, tissue optics, diffuse scattering, oxygenation, tissue chromophores, self-calibrating approach, ratiometric approach

## Abstract

We report on the comparative analysis of self-calibrating and single-slope diffuse reflectance spectroscopy in resistance to different measurement perturbations. We developed an experimental setup for diffuse reflectance spectroscopy (DRS) in a wide VIS-NIR range with a fiber-optic probe equipped with two source and two detection fibers capable of providing measurements employing both single- and dual-slope (self-calibrating) approaches. In order to fit the dynamic range of a spectrometer in the wavelength range of 460–1030 nm, different exposure times have been applied for short (2 mm) and long (4 mm) source-detector distances. The stability of the self-calibrating and traditional single-slope approaches to instrumental perturbations were compared in phantom and in vivo studies on human palm, including attenuations in individual channels, fiber curving, and introducing optical inhomogeneities in the probe–tissue interface. The self-calibrating approach demonstrated high resistance to instrumental perturbations introduced in the source and detection channels, while the single-slope approach showed resistance only to perturbations introduced into the source channels.

## 1. Introduction

Diffuse reflectance spectroscopy (DRS) is an optical technique that allows the evaluation of tissue biochemistry and microstructure for a number of applications including brain hemodynamics [1] also called fNIRS, diagnostics of breast tumor margins [2,3] and treatment monitoring [4], skin cancer diagnostics [5,6,7], evaluating the scar severity and therapeutic response of keloid [8], and diagnostics of tumor margins in the oral cavity (head and neck cancer) [9], lung [10], liver [11,12,13], and colon [14,15]. A number of potential applications have also been reported, such as diagnostics of thyroid [16] and adipose tissue [17] and the identification of neurovascular bundles. The DRS principle is based on delivering broadband light to the biotissue and registering the backscattered light at a specified distance. The detected signal contains information about scattering (related to the microstructure of the tissue) and absorption (related to its biomolecular composition). Due to the strong dependence of absorption coefficients of different chromophores (oxy- and deoxyhemoglobin, melanin, lipids, water, etc.) on the wavelength, one can reconstruct their concentrations in tissue by analyzing the extinction of the light spectrum between the source and detector.

The DRS probing spectral range is selected depending on the absorption spectra of the studied chromophores and the desired probing volume in tissue. For example, the concentrations of oxy- and deoxyhemoglobin in superficial tissues can be reconstructed using the visible (usually 500–600 nm) spectral range, while for deeper probing it is reasonable to use the range of 700–900 nm due to higher light penetration depth. The NIR range is also used to assess water and lipid content [18], while the wider VIS-NIR range can be used for analysis of collagen and elastin content [8]. Currently, VIS-NIR spectroscopy has been applied in several works and has shown higher potential in comparison with VIS or NIR spectroscopy separately [14,19,20], because it allows the reconstruction of concentrations of a larger set of tissue chromophores and/or obtaining a higher accuracy [18,21].

If tissue optical properties vary with depth, DRS in the VIS-NIR range can be applied to assess chromophore concentrations in different tissue layers using differences in sensitivity depths of the VIS and NIR spectrum regions. This approach was successfully applied to assess skin hemoglobin concentrations in the dermis and lower dermis [22].

The results of the reconstruction of tissue chromophores in DRS have the following keys to success: (1) the applied light transport model should be realistic enough to correctly describe light attenuation from source to detector; (2) the reconstruction procedure should have a good convergence; (3) instrumental characteristics, such as source spectral brightness, detector spectral sensitivity, transient characteristics of source and detector fiber channels, and optical contact between the DRS probe and tissue should be taken into account. In the present study we concentrate mainly on the last issue because incorrect consideration of the instrumental characteristics can lead to significant errors in the reconstructed values even with an appropriate light transport model and a valid reconstruction technique. This aspect is especially essential in broadband measurements, in particular VIS-NIR, due to possible light dispersion in the instrumental part and strong differences in light attenuation in tissue in different spectral ranges, and, therefore, a need for adjusted compensation. 

Different strategies are applied in DRS to correctly account for the instrumental characteristics depending on the measured data type. For example, investigation of hemodynamics based on the measurement of relative changes in hemoglobin concentrations in time can be implemented using a simple single source-detector distance (SDD) approach with a single source-detector pair. The resulting equation for relative changes in hemoglobin concentrations in time allows for the exclusion of instrumental characteristics [23]. Absolute measurements of chromophore concentrations in a single SDD configuration are usually accompanied by calibration measurements with a tissue phantom with known absorption and scattering characteristics or a reflection standard [24]. However, if instrumental characteristics vary in time (for example, source spectral brightness may significantly vary in lamp sources), a calibration procedure should be applied periodically, which is not convenient or even impossible during continuous biomedical examination. Continuous calibration measurements can be provided with the help of an additional source-detector channel with a reflection standard at the tips of source and detection optical fibers [25].

Two SDDs with a single source and two detectors or a single detector and two sources allow the assessment of effective light attenuation *µ_eff_* in tissue by taking a ratio between the detected signals obtained at different SDDs. In this ratiometric approach, also called single-slope measurement, most of the instrumental characteristics are excluded in the final equation for *µ_eff_*, which yields a more accurate assessment of tissue chromophore content in comparison with the single-distance approach [26]. Multiple sources at a single detector or multiple detectors at a single source are used to increase the precision of the extinction coefficient extraction. However, the effect of instrumental function is not completely eliminated in this approach.

**Figure 1:**
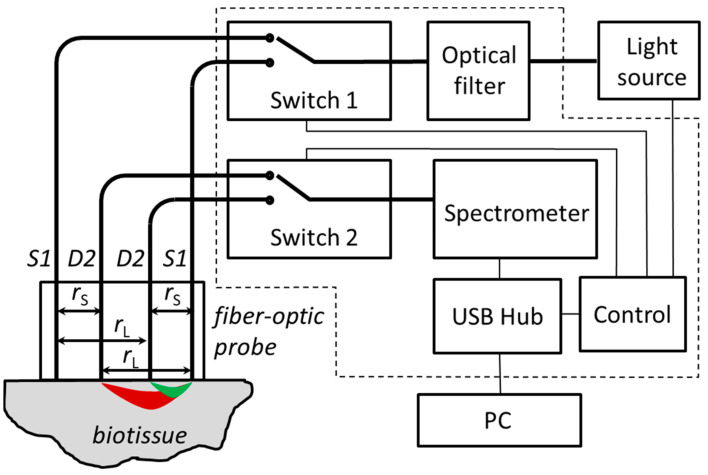
Schematic of experimental setup with self-calibrating fiber-optic probe. S1,2: the source fibers; D1,2: the detection fibers; areas of effective sensitivities for short (*r_S_*) and long (*r_L_*) SDDs are marked with green and red colors, respectively. Bold lines indicate optical fibers, thin lines indicate electrical connections. Elements within the dashed frame are placed in a single housing.

A possible solution to compensate for more instrumental contributions is a self-calibrating technique suggested in [27]. The idea is based on symmetrical multi-distance measurements; at least four measurements at each wavelength with two sources and two detectors with a symmetrical configuration (Figure 1) should be provided to obtain calibration-free characterization of the studied tissue. This probe demonstrated more reliable data on the optical properties of tissue and higher long-term stability compared to standard DRS configuration due to a reduction in instrumental errors.

In addition to insensitivity toward instrumental effects, the self-calibrating approach is less sensitive to the changes in optical coupling between the optical probe and tissue. The last advantage is tightly connected with the differences in sensitivity to superficial or deeper chromophores in different approaches: the traditional single-measurement approach has a banana-shaped sensitivity function [28] with maxima near source– and detector–tissue interfaces, while the self-calibrating technique is relatively more sensitive to deeper tissues [29,30]. This finding makes the self-calibrating approach very attractive in its application for studies of brain activity in the NIR spectral region [31]. Single-distance or single-slope approaches register primarily photons backscattered from the scalp and skull masking the brain hemodynamics, and the traditional increase in SDD does not provide any significant benefit, since the maximum sensitivity remains near the source– and detector–tissue contacts. The self-calibrating approach was applied for diagnostics of breast tumors with a more sophisticated probe including 16 continuous-wave (CW) sources at 690 nm and 830 nm and 8 detectors located symmetrically [32]. Multiple sources and detectors allow obtaining a signal averaged over a large tissue volume resulting in more robust data on the oxygenation of tumor tissue [33].

The main drawback of all pure CW optical diffuse measurements is related to the difficulty of separating absorption *µ_a_* and reduced scattering $\mu_{s}^{\prime }$ coefficients which are included in the expression for the effective extinction coefficient of diffuse light as a product:

(1) $$\mu_{eff}=\sqrt{3\mu_{a}\left(\mu_{a}+\mu_{s}^{\prime }\right)}.$$

Employing the reduced scattering coefficient values from the literature may cause significant errors in absolute measurements of chromophore concentrations due to tissue-to-tissue variations in reduced scattering values [34]. Additional frequency-domain (FD) measurements employing high-frequency modulation of probing light intensity at two or more wavelengths allow assessing reduced scattering directly at these wavelengths. Assuming a power law or a linear wavelength dependence of reduced scattering coefficient (the latter approach is reasonable in NIR where the decrease in $\mu_{s}^{\prime }\left(\lambda \right)$ dependence is slow), one can estimate it within the whole spectrum measured by CW DRS, which results in higher precision of chromophores reconstruction [35] (steady-state and frequency-domain (SSFD) reflectance measurements). SSFD measurements at two wavelengths were also successfully applied in combination with a self-calibrating approach for studying human brain hemodynamics [36,37].

The FD technique is applied successfully for the NIR spectral range for large SDDs that ensure a sufficient phase shift of a photon density wave propagated from source to detector. However, at large SDDs (approx. more than 5 mm), VIS measurements are restricted by high attenuation of light in biotissue. Smaller SDDs (<5 mm) need higher modulation frequency to register the phase shift that is hard to implement technically. In this connection, DRS in VIS range 500–600 nm is usually performed with the CW technique alone [26]. In the joint VIS-NIR range, the reduced scattering spectrum $\mu_{s}^{\prime }\left(\lambda \right)$ has a more sophisticated behavior and can be approximated by a sum of Rayleigh-scattering and Mie-scattering components [18], since Rayleigh scattering is assumed to dominate in the UV-blue optical range, while Mie scattering prevails in NIR:

(2) $$\mu_{s}^{\prime }\left(\lambda \right)=a\left[f{\left(\frac{\lambda }{\lambda_{0}}\right)}^{-4}+\left(1-f\right){\left(\frac{\lambda }{\lambda_{0}}\right)}^{-b}\right]$$

Here the parameter *a* is the reduced scattering coefficient at $\lambda_{0}$ = 500 nm, *f* is the fraction of Rayleigh scattering with λ^−4^ dependence that is described by the first term in the brackets, and *b* is the power index of Mie scattering wavelength dependence described by the second term. Parameters *a*, *b*, and *f* can be assessed along with unknown tissue chromophores composition from the fitting of an experimentally obtained reflected spectrum by a model function. This approach has been applied in VIS-NIR DRS for a simplified $\mu_{s}^{\prime }\left(\lambda \right)$ dependence [38].

In this paper, we present a comparative analysis of the sensitivity of the dual- and single-slope approaches in DRS to various perturbations than can occur in the course of measurements. The analysis is based on the assessment of the changes in the reconstructed effective extinction coefficient spectrum in response to the introduced instrumental distortion. The study is performed using a custom-built experimental setup for VIS-NIR DRS with a fiber-optic probe employing a self-calibrating approach. To the best of our knowledge, this is the first application of a self-calibrating approach for ultra-wideband 460–1030 nm (VIS-NIR) DRS. The problem that arises is the significant difference in absorption coefficients in VIS and NIR spectral regions, leading to difficulties in detecting both regions simultaneously with a single spectrometer at different SDDs with a large enough signal-to-noise ratio. In the proposed experimental setup, we solved this problem by applying individual exposure times for small and large SDDs in order to fit the whole received spectra for both SDDs in the spectrometer dynamic range. The reconstruction of tissue optical parameters is proposed via a minimization in the difference between the effective extinction coefficient μ*_eff_*, which is evaluated from DRS measurements using simplified light diffusion theory, and the expected model coefficient *µ_eff_* calculated with Equation (1). The absorption coefficient in Equation (1) is assumed to be a linear combination of basic biotissue chromophores absorption spectra, while the reduced scattering coefficient is described by Equation (2). The developed experimental setup has been tested for resistance toward different instrumental perturbations including the bending of optical fibers, installing an additional attenuator in an individual channel, and modifying the probe–tissue interface. The results for the self-calibrating approach were compared to those for the single-slope measurements.

## 2. Materials and Methods

### 2.1. Evaluation of Extinction Coefficient with Self-Calibrating Approach Measurements

Propagation of light between source and detector in biological tissue is well described by the Radiative Transfer Equation (RTE) [39], employing absorption *µ_a_* and scattering *µ_s_* indices and a scattering phase function as tissue optical properties. There exists no general analytical solution to this equation, however, for a number of applications with SDD exceeding several light transport lengths, the RTE can be reduced to the diffuse equation, which has an analytical solution for homogeneously scattering and absorbing media. In the frame of this approach, a photon fluence rate generated by a point light source with unit power in the infinite medium (Green’s function) is defined by equation [40]:

(3) $$\phi \left(r\right)=\frac{3\left(\mu_{a}+\mu_{s}^{\prime }\right)}{4\pi r}\exp\left(-\mu_{eff}r\right)$$

where *r* is the distance from the source and $\mu_{eff}$ is defined by Equation (1) via the absorption coefficient *µ_a_* and reduced scattering coefficient $\mu_{s}^{\prime }$. Under certain assumptions (neglecting medium boundary conditions and radiating patterns of source and detection fibers), Equation (3) can be used to characterize light intensity in the detection fiber *Dj* located at distance $r_{kj}$ from the source fiber *Sk* (*j*,*k* = 1,2) in the configuration shown in Figure 1. Following these assumptions, the DRS signal registered by a spectrometer at the specific wavelength can be written in the following form:

(4) $$I_{kj}=\frac{3\left(\mu_{a}+\mu_{s}^{\prime }\right)AS_{k}AD_{j}I_{0}\eta }{4\pi r_{kj}^{}}E_{kj}\exp\left(-\mu_{eff}r_{kj}\right)$$

where $I_{0}$ is a light source intensity; $AS_{k}$ and $AD_{j}$ are the transient characteristics of DRS device parts describing the propagation of light from the light source to a contact of the source fiber *k* with the biotissue and from the biotissue contact with the detection fiber *j* to the spectrometer, respectively; *η* is the spectrometer sensitivity; and $E_{kj}$ is the corresponding spectrometer exposure time.

The ratio of signals received with a common source *k* and two detectors *j* = 1,2 having different SDDs (known as single-slope configuration) carries information about the medium extinction coefficient and excludes most of instrumental characteristics (source transient characteristics, source brightness, and spectrometer sensitivity):

(5) $$\frac{I_{k1}}{I_{k2}}=\frac{AD_{1}r_{k2}^{}E_{k1}}{AD_{2}r_{k1}^{}E_{k2}}\exp(-\mu_{eff}(r_{k1}-r_{k2}))$$

Typically an assumption is made that both detection channels have equal transient characteristics, i.e., $AD_{1}=AD_{2}$. In this case, one can evaluate the extinction coefficient following Equation (5) and using the ratio $I_{11}/I_{12}$ of the spectra measured by the system depicted in Figure 1 with source S1 and two detectors D1 and D2 (abbreviated as S1D1D2) as:

(6) $$\mu_{eff}=\frac{1}{r_{12}-r_{11}}\ln\left[\left(\frac{I_{11}}{I_{12}}\right)\left(\frac{E_{12}}{E_{11}}\right)\frac{r_{11}^{}}{r_{12}^{}}\right]$$

and using the ratio $I_{22}/I_{21}$ of the spectra measured with source S2 and two detectors D1 and D2 (abbreviated as S2D1D2) as:

(7) $$\mu_{eff}=\frac{1}{r_{21}-r_{22}}\ln\left[\left(\frac{I_{22}}{I_{21}}\right)\left(\frac{E_{21}}{E_{22}}\right)\frac{r_{22}^{}}{r_{21}^{}}\right]$$

However, if the assumption $AD_{1}=AD_{2}$ is incorrect, the extinction spectrum calculated by Equations (6) or (7) contains an error. In this case, for symmetrical measurements when $r_{11}=r_{22}=r_{S}$ and $r_{21}=r_{12}=r_{L}$, Equation (5) yields:

(8) $$\left\{\begin{array}{l} \begin{array}{l} \begin{array}{l} \frac{I_{11}}{I_{12}}=\frac{AD_{1}r_{L}^{}E_{11}}{AD_{2}r_{S}^{}E_{12}}\exp(\mu_{eff}\left(r_{L}-r_{S}\right))\\ \frac{I_{21}}{I_{22}}=\frac{AD_{1}r_{S}^{}E_{21}}{AD_{2}r_{L}^{}E_{22}}\exp(-\mu_{eff}\left(r_{L}-r_{S}\right)) \end{array} \end{array} \end{array}\right.$$

and the effective extinction coefficient can be derived in the form that excludes both source and detector transient characteristics:

(9) $$\mu_{eff}=\frac{1}{2\left(r_{L}-r_{S}\right)}\ln\left[\left(\frac{I_{11}I_{22}}{I_{12}I_{21}}\right)\left(\frac{E_{12}E_{21}}{E_{11}E_{22}}\right)\frac{r_{S}^{2}}{r_{L}^{2}}\right]$$

Equation (9) represents a self-calibrating (or dual-slope) approach, employing four source-detector measurements: S1-D1, S1-D2, S2-D1, S2-D2 (abbreviated as S1D1S2D2). One can note that expression (9) is an average value of the extinction coefficients obtained by Equations (6) and (7).

### 2.2. Reconstruction of Skin Chromophores and Scattering from $\mu_{eff}\;\text{Spectrum}$

In this study we focus on the characterization of human skin scattering and absorption spectra. Multiplicative combinations of absorption coefficients and reduced scattering coefficients in expression (1) for the extinction coefficient makes their separation from the continuous wave measurements difficult, in contrast to frequency-domain or time-domain techniques. In our study, in order to separate *µ_a_* from $\mu_{s}^{\prime }$ based on Equation (1) and the extinction coefficient spectrum experimentally obtained with Equations (6), (7), or (9), depending on the employed measurement scheme, we make several empirical assumptions.

The absorption coefficient $\mu_{a}$ is considered as a weighted sum of the absorption spectra of basic skin chromophores. In our consideration we limit their set to melanin, blood, water, and “dry matter”, assuming that the contribution of other chromophores in the range of 460–1030 nm is negligible:

(10) $$\mu_{a}(\lambda )=C_{water}*\mu_{a}^{water}(\lambda )+C_{mel}*\mu_{a}^{mel}(\lambda )+\;\left[C_{blood}*\left(StO_{2}\;*\mu_{a}^{oxy}(\lambda )+\left(1-StO_{2}\right)*\mu_{a}^{deoxy}(\lambda )\right)\right]+\mu_{a}^{dry}$$

where the spectra of $\mu_{a}^{HbO_{2}}$, $\mu_{a}^{Hb}$, and $\mu_{a}^{mel}$ are taken from [41,42]. Absorption of “dry matter” is taken as a wavelength independent $\mu_{a}^{dry}$. A reduced scattering coefficient spectrum is taken according to Equation (2) with the Rayleigh fraction which approximates scattering at small wavelengths and Mie fraction which dominates in NIR. The contribution of lipids to the absorption coefficient is not considered in this study since the SDD in the described system is limited by 4 mm and the measurements described below have been performed on human palm with low lipid content. The parameters of oxygen saturation $StO_{2}$ concentration of different chromophores,—$C_{water}$, $C_{mel}$, $C_{blood}$, $\mu_{a}^{dry}$—together with the scattering parameters a, *b*, and *f* from Equation (2), can be determined by fitting the experimentally obtained extinction spectrum $\mu_{eff}$ with a combination of empirical dependencies (10) and (2) substituted into Equation (1). This optimization problem was solved numerically by finding the parameters vector $\begin{array}{c} K=[\;C_{blood},\;StO_{2},\;C_{water},\mu_{a}^{dry},a,\;b,f \end{array}]$ using lsqcurvefit MATLAB function within the wavelength range of 460–1030 nm assuming $C_{\mathrm{mel}}$ to be a constant equal to 0.005. This solution can be derived using Equation (9) in the case of the self-calibrating approach as:

(11) $$K=\underset{K}{\mathrm{argmin}}\underset{\lambda }{\sum }{\left(\sqrt{3\mu_{a}\left(K\right)\left(\mu_{a}\left(K\right)+\mu_{s}^{\prime }\left(K\right)\right)}-\frac{1}{2\left(r_{L}-r_{S}\right)}\ln\left[\left(\frac{I_{11}I_{22}}{I_{12}I_{21}}\right)\left(\frac{E_{12}E_{21}}{E_{11}E_{22}}\right)\frac{r_{S}^{2}}{r_{L}^{2}}\right]\right)}^{2}$$

or in the case of single-slope approach in S1D1D2 mode using Equation (6) as:

(12) $$\begin{array}{c} K \end{array}=\underset{K}{\mathrm{argmin}}\sum_{\lambda }{\left(\sqrt{3\mu_{a}\left(K\right)\left(\mu_{a}\left(K\right)+\mu_{s}^{\prime }\left(K\right)\right)}-\frac{1}{r_{L}-r_{S}}\ln\left[\left(\frac{I_{11}}{I_{12}}\right)\left(\frac{E_{12}}{E_{11}}\right)\frac{r_{S}}{r_{L}}\right]\right)}^{2},$$

or in S2D1D2 mode using Equation (7) as:

(13) $$\begin{array}{c} K \end{array}=\underset{K}{\mathrm{argmin}}\sum_{\lambda }{\left(\sqrt{3\mu_{a}\left(K\right)\left(\mu_{a}\left(K\right)+\mu_{s}^{\prime }\left(K\right)\right)}-\frac{1}{r_{L}-r_{S}}\ln\left[\left(\frac{I_{22}}{I_{21}}\right)\left(\frac{E_{21}}{E_{22}}\right)\frac{r_{S}}{r_{L}}\right]\right)}^{2}.$$

During the optimization procedure the following limitations were set on the extracted variables: $StO_{2}$ varies in the physiological range of [0, 1], *a* varies in the range of [0.5, 10] mm^−1^, b varies in the range of [0, 3], *f* varies in the range of [0, 1], volume fractions *C_blood_* and *C_water_* vary within [0, 1], and $\mu_{a}^{dry}$ varies within [0, ∞]. Lower and upper limits for the parameters *a* and *b* are determined according to the reported data on the range of the skin reduced scattering coefficient at $\lambda_{0}=500\;\mathrm{nm}$ and the corresponding power index values [18,43]. Since $\mu_{a}^{mel}(\lambda )$ and $\mu_{s}^{\prime }(\lambda )$ both monotonously decrease with the wavelength, high uncertainty arises in the joint reconstruction of the parameters of $C_{\mathrm{mel}}$, *a*, *b*, and *f*; therefore, $C_{\mathrm{mel}}$ was chosen as a constant value.

### 2.3. Experimental DRS Setup

An experimental DRS setup with a self-calibrating fiber-optic probe was constructed in accordance with the scheme shown in Figure 1. Radiation from Fiber-Coupled Xenon (SLS205, Thorlabs, Newton, NJ, USA) broadband 240–1200 nm light source was used for tissue probing. The source has a mechanical shutter driven by a TTL pulse from the Control unit Arduino Uno (Arduino, Scarmagno, Italy). It has rather high spectral radiance both in VIS and NIR spectral regions in comparison with those traditionally applied in VIS-NIR spectroscopy tungsten halogen lamps which have low spectral radiance at wavelengths below 600 nm. Probing light passes through BS-8 (Zapad Pribor, Moscow, Russia) absorption filter cutting UV light below 380 nm placed in the fiber-optic FOFMS/M (Thorlabs Inc., Newton, NJ, USA) filter holder. A UV filter is used to prevent possible negative effects on biological tissue and solarization of the probe and switch optical fibers. Spectrally corrected light passes through 1 × 2 fiber-optical switch 1 (Piezosystem Jena GmbH, Jena, Germany) which selects the *S*1 or *S*2 source fiber of the probe to illuminate biological tissue. Diffusively scattered light is detected by detection fibers *D*1 and *D*2, one of which is selected by a 1 × 4 switch 2 (Piezosystem Jena GmbH, Jena, Germany) to deliver light to spectrometer Maya 2000 PRO (Ocean Optics, Orlando, FL, USA). Switch 2 uses only two outputs for the applied optical probe which has only two detection fibers. Both switches are driven by the control unit. The Maya spectrometer has rather high sensitivity in NIR up to 1100 nm which covers water and lipid absorption bands near 980 nm and has high linearity due to the applied calibration which is essential for the application of a self-calibrating approach. The spectrometer exposure times are set individually for small and large SDDs to fit the spectrometer dynamic range. In the current in vivo and phantom studies, the exposure times were set equal to $E_{21}=E_{12}=80$ ms for long SDD $r_{L}$= 4 mm and $E_{11}=E_{22}=15$ ms for short SDD $r_{S}$=2 mm. For each source–detector pair, the detected signal is averaged over several subsequent measurements in order to increase signal-to-noise ratio with the following subtraction of a dark signal obtained at the closed source shutter with the same exposure time and averaging. For the in vivo studies, the averaging number is taken from the ratio T/$E_{21}$ or T/$E_{11}$ where T is a heartbeat period. The resulting spectra *I*_11_, *I*_12_, *I*_21_, and *I*_22_ are stored at PC for further analysis described below in Section 2.3.

Domestically designed probe housing is made of black photopolymer resin Anycubic Basic (HONGKONG Anycubic Technology Co., Limited, Hong Kong, China) by 3D printing on a Phrozen Shuffle 2019 (Hsinchu City, Taiwan) printer and has 6 × 8 mm^2^ area of the probe–tissue interface. Two 400 µm source fibers and two 200 µm detection fibers with 0.22 NA (Thorlabs, Newton, NJ, USA) were placed in line inside the probe housing at a 2 mm distance between neighboring fibers, which results in short and long SDDs of *r_S_* = 2 mm and *r_L_* = 4 mm, respectively.

The fiber-optic probe is equipped with a mechanical pressure control unit. The pressure was set equal to 12.7 kPa in all studies.

The DRS system is fully automated by a JAVA code operated with a source shutter, spectrometer, and fiber switches with the help of a control unit. Full acquisition time was about 6 s for the abovementioned exposure times and a heartbeat period of 1 s.

### 2.4. Instrumental Perturbations in Phantom and In Vivo DRS Studies

The developed experimental setup was tested on a silicone-based biotissue phantom employed as a reference standard for DRS measurements in [26] and on a palm of a healthy volunteer from the group of the researchers. In both series of experiments, various types of instrumental perturbations were applied to a developed DRS setup in order to compare the stability of the self-calibrating and single-slope approaches, including installing an additional attenuator in an individual channel, bending the optical fiber, and modifying the probe–tissue interface (Table 1).

Installing additional fibers (labeled as D1L and S1L in Table 1) with a smaller core diameter of 105 μm (compared to 200 and 400 μm fibers used in the setup) in an individual channel simulates possible losses in fiber-optic contacts between different instrumental parts: the light source, detector, optical switches, and fiber-optic probe. Curving a probe fiber (labeled as D1C and S1C in Table 1) into a 50 mm radius ring simulates the fiber curvature occurring in the course of a medical procedure when a fiber-optic probe examines different tissue localizations and fibers are randomly bent. Modification of the probe–tissue interface with plastic page stickers (series NEON, BRAUBERG, Frankfurt, Germany) with different colors (blue, green, and pink labeled as D1B and S1B, D1” and S1G, and D1P and S1P, respectively) simulates random biotissue surface inhomogeneities that are always present in biological tissue examinations.

**Figure 2:**
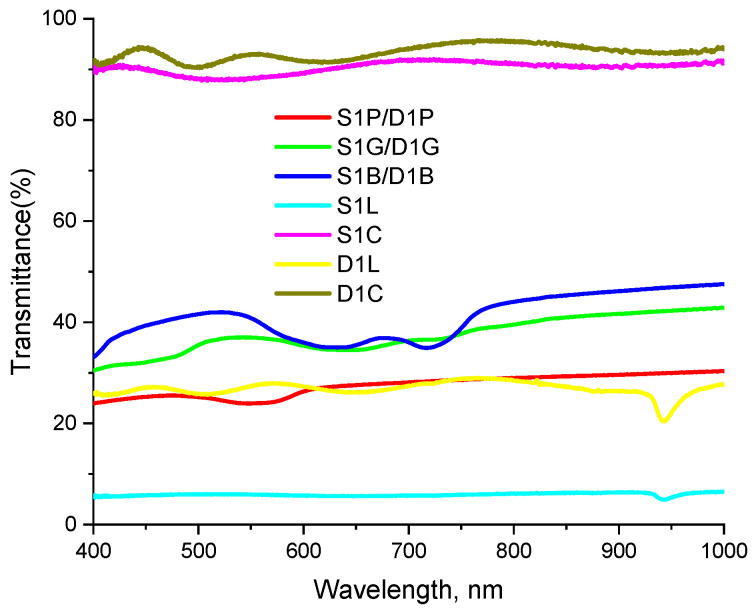
Transfer functions of the studied perturbations (the ratio of the measured spectrum with the perturbation introduced to that in the absence of the perturbation). Perturbations are indicated in accordance with Table 1.

In order to quantify the spectral effects of all the studied perturbations, we measured the spectral transfer functions of the introduced perturbation (the ratio of the measured spectrum with the perturbation introduced to that in the absence of the perturbation). The measurement results are shown in Figure 2 and demonstrate that most of the considered perturbations lead to spectrum shape distortion which may potentially lead to errors in reconstruction of physiological properties from the DRS measurements.

The colored stickers naturally feature transmission bands corresponding to their visible colors. Note a significant difference in the transmittance coefficient for perturbations S1L and D1L consisting in the insertion of an additional fiber to the source or the detection channel, respectively, that originates from the different mismatch between the diameters of the original fibers and the inserted fiber. For each perturbation, DRS measurements were repeated 3 times; before each measurement the position of a probe was slightly changed by removing and then replacing the probe. Unperturbed measurements were repeated 6 times: 3 times before applying perturbations and 3 times after.

The perturbations applied to source S2 and detector D2 are not listed in Table 1 because they provide similar results to the perturbations applied to S1 and D1 for reasons of symmetry (Figure 1).

### 2.5. Calculation of Extinction Spectra Variations

Various instrumental perturbations listed in Table 1 result in different deviations in the extinction spectrum calculated by Equations (6), (7), or (9). These deviations are quantified as a root mean square deviation (RMSE) of the extinction spectrum values $\mu_{eff}^{P,m}\left(\lambda \right)$ evaluated under particular perturbation in a single experiment from the initial $\mu_{eff}^{INIT}$ extinction spectrum measured without perturbations:

(14) $$\Delta \mu_{eff}^{P,m}=\sqrt{\frac{\sum_{i=1}^{N_{\lambda }}{\left(\mu_{eff}^{P,m}\left(\lambda_{i}\right)-\mu_{eff}^{INIT}\left(\lambda_{i}\right)\right)}^{2}}{N_{\lambda }}}$$

where $\lambda_{i}$ is the *i*-th wavelength, $i=1\ldots N_{}$; *P* is the perturbation index listed in Table 1, and *m* is the measurement number with the particular perturbation $m=1\ldots N_{P}$. For perturbed measurements, $N_{P\neq INIT}=3$, and for unperturbed measurements, $N_{P=INIT}=6$. The initial extinction spectrum $\mu_{eff}^{INIT}\left(\lambda_{i}\right)$ is calculated as an average value for each wavelength over 6 measurements provided without any perturbations. To quantify deviations caused by perturbations of type *P*, an average value over $\Delta \mu_{eff}^{P,m}$ is calculated:

(15) $$\Delta \mu_{eff}^{P}=\frac{\sum_{m=1}^{N_{P}}\Delta \mu_{eff}^{P,m}}{N_{P}}$$

Variations of reconstructed tissue chromophore concentrations and scattering properties obtained for in vivo measurements were calculated in the same way.

## 3. Results

### 3.1. Phantom DRS Measurements

Several DRS phantom measurements have been performed with and without the instrumental perturbations indicated in Table 1.

**Figure 3:**
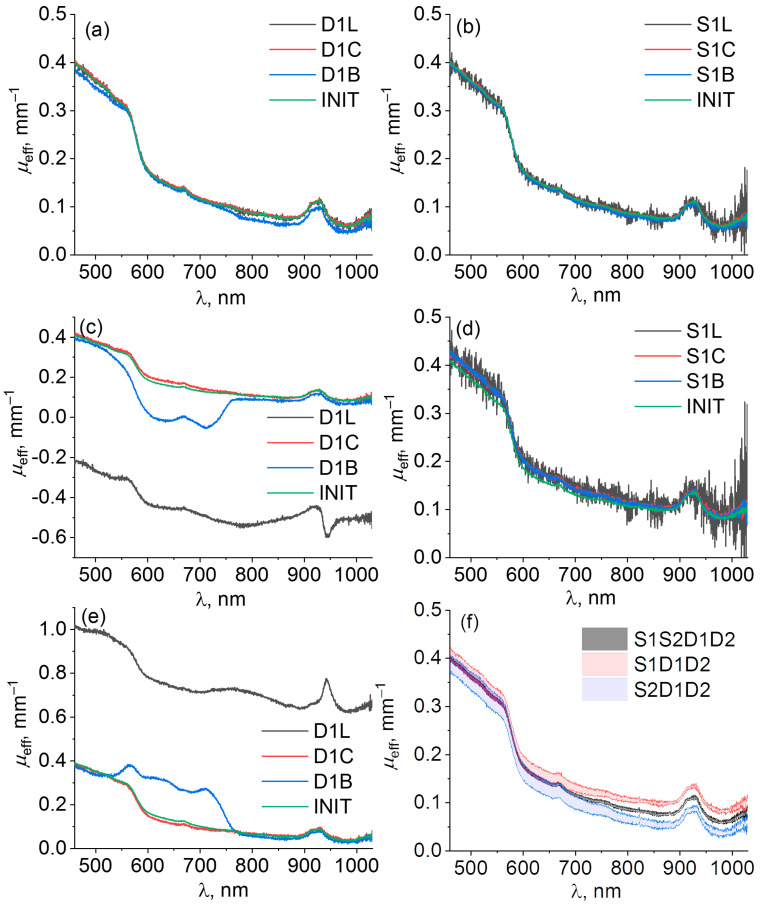
Spectra of $\mu_{eff}^{P,m}$ of a biotissue phantom calculated with self-calibrating approach S1S2D1D2 (**a**,**b**) and single-slope approach in configurations S1D1D2 (**c**,**d**) and S2D1D2 (**e**) for different kinds of detector (left column) and source (right column) perturbations listed in Table 1. Index m indicates individual measurement under particular perturbation P. Comparison of $\mu_{eff}^{INIT}$ spectra (**f**) obtained by three approaches S1S2D1D2 (gray), S1D1D2 (red), and S2D1D2 (blue) for unperturbed measurements shown as mean with confidence bounds.

Figure 3 shows the examples of extinction spectra $\mu_{eff}^{P,m}$ of a silicone phantom calculated using the single-slope approach in S1D1D2 and S2D1D2 configurations and the calibration-free approach in S1S2D1D2 configuration for different kinds of source (S1L, S1C, S1B) and detector (S1L, S1C, S1B) perturbations, as well as for unperturbed data. As one can see from Figure 3a,b,f, all extinction spectrum curves calculated using the self-calibrating approach are close to each other, which indicates a high resistance to instrumental perturbations introduced to source or detector channels (Figure 3a,b). In contrast, the single-slope approach demonstrates resistance only to source perturbations (Figure 3(d)), while perturbations introduced to the detector channel lead to significant variations in $\mu_{eff}^{}$ (Figure 3c,e). It can be seen from Figure 3c,e that if a perturbation *P* results in an increase in $\mu_{eff}^{P}$ values calculated by S1D1D2 data, the value of $\mu_{eff}^{P,m}$ calculated by S2D1D2 data decreases. For example, the absorption band of the blue sticker (see Figure 2) employed in perturbation D1B manifests by the deformation of the $\mu_{eff}$ spectrum reconstructed by single-slope measurements in S1D1D2 and S2D1D2 configurations in opposite ways (Figure 3(c) and 3(e), respectively) according to Equations (6) and (7). The introduction of loss perturbation D1L appears as a negative (Figure 3c) or positive (Figure 3e) shift in the reconstructed $\mu_{eff}$ spectrum together with a variation around 950 nm which is determined by the transmission peak in the transfer function of this perturbation (Figure 2). The fiber curving perturbation D1C provides minimal variations in $\mu_{eff}$ spectrum since it has a transmittance close to 100% (Figure 2). The increase in the noise level in Figure 3b,d for S1L perturbation is caused by a drop in light intensity, while the shape of the extinction spectra does not change in both S1D1D2 and S1S2D1D2 cases.

Figure 3f demonstrates variations in the extinction spectra of the uniform silicone phantom for repeated unperturbed measurements. Variations for the single-slope configurations are higher than those for the self-calibrating approach. It should also be noted that red and blue curves corresponding to $\mu_{eff}$ calculated by the single-slope approach in the two configurations S1D1D2 and S2D1D2, respectively, are positioned above and below the gray curve for $\mu_{eff}\;$ recovered by the self-calibrating approach, which is associated with non-identical transient characteristics of the detection channels D1 and D2.

### 3.2. In Vivo DRS Measurements of Human Skin

**Figure 4:**
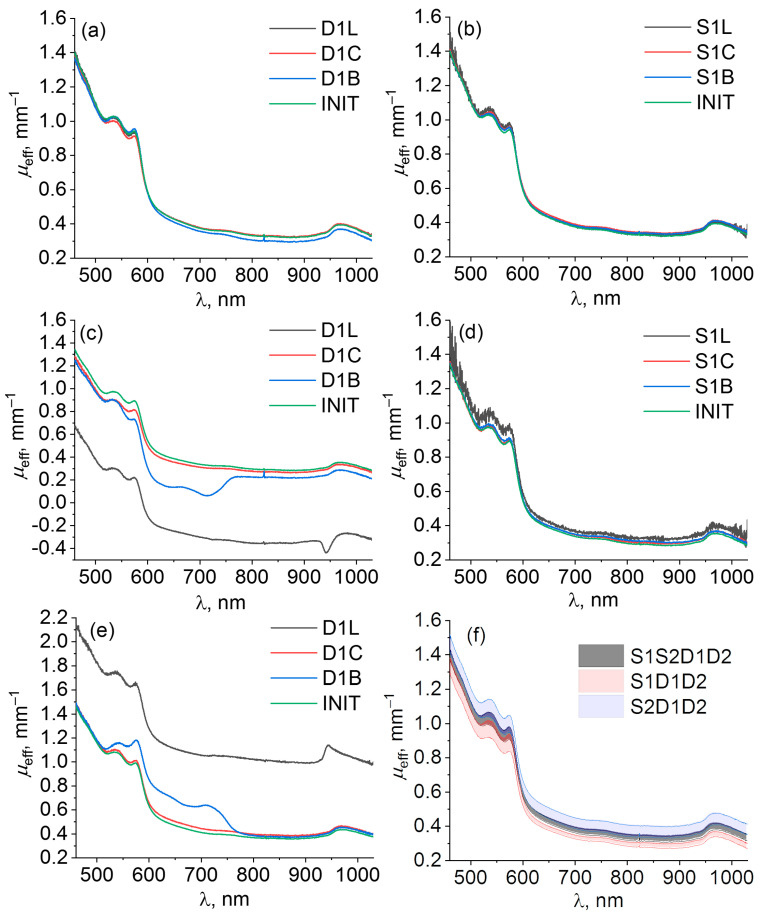
Examples of $\mu_{eff}^{P,m}$ spectra of a human palm calculated with self-calibrating approach S1S2D1D2 (**a**,**b**), and single-slope configurations S1D1D2 (**c**,**d**), and S2D1D2 (**e**) for different kinds of detector (left column) and source (right column) perturbations described in Table 1. Index m indicates individual measurements under particular perturbation P. Comparison of $\mu_{eff}^{INIT}$ spectra (**f**) obtained by three approaches S1S2D1D2 (gray), S1D1D2 (red), and S2D1D2 (blue) for unperturbed measurements shown as mean with confidence bounds.

In vivo DRS measurements demonstrate almost similar results to the phantom studies (Figure 4). High resistance of the self-calibrating approach to both source (Figure 4b) and detector (Figure 4a) instrumental perturbations is observed for this case, whereas the single-slope approach has resistance only to source perturbations (Figure 4d), while detector perturbations can lead to a significant corruption of the reconstructed extinction spectrum (Figure 4c,e) such as those observed for the silicone phantom measurements (Figure 3c,e).

In contrast to the results of phantom studies, the extinction coefficient in in vivo studies calculated for unperturbed (INIT) measurements (Figure 4f) demonstrate similar variations in self-calibrating and the single-slope approaches. This can be explained by the spatial variations of palm biotissue optical properties that are much higher than the variations of a homogeneous tissue phantom.

**Figure 5:**
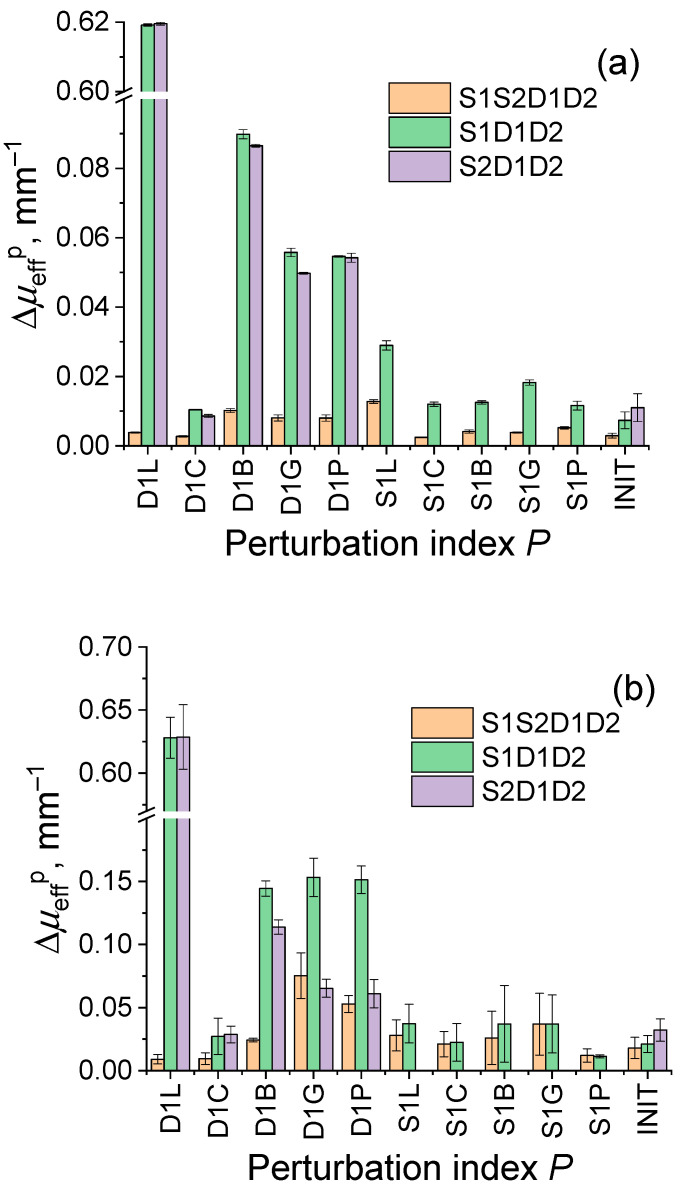
Deviations in extinction coefficient $\Delta \mu_{eff}^{P}$ spectra calculated for different types of perturbations by Equation (15) using single-slope approach in configurations S1D1D2 and S2D1D2 and self-calibrating approach S1S2D1D2 in phantom (**a**) and in vivo (**b**) studies. Error bars show deviations in $\Delta \mu_{eff}^{P,m}$ values.

Figure 5 provides the comparison of the average deviations in the extinction coefficient $\Delta \mu_{eff}^{P}$ for different types of perturbations in the studies of a biotissue phantom and a human palm. In Figure 5a the deviations in reconstructed extinction coefficient $\Delta \mu_{eff}^{P}$ for all types of perturbations in phantom studies are summarized. It can be seen from the diagram that deviations in the extinction coefficient calculated by the self-calibration approach are smaller than those calculated by the single-slope approach for all types of perturbations. 

For unperturbed measurements, deviations in the extinction coefficient are 0.003 mm^−1^ for the self-calibration approach, and 0.007 and 0.011 mm^−1^ for the single-slope technique in two configurations, respectively. For all types of perturbations, the deviation of the extinction coefficient calculated by the self-calibration approach does not exceed 0.013 mm^−1^, while deviations in this value calculated using the single-slope approach exceed 0.6 mm^−1^ for the loss perturbations in detection channels. It also can be seen from this plot that the deviations in the extinction coefficient caused by perturbations in the source channel are smaller than those applied to the detection channel. Figure 5b shows the values of the deviations for the in vivo measurements. Similar to Figure 5a, detector perturbations (D1L, D1C, D1B, D1G, D1P) lead to large values of deviations in the extinction coefficient calculated using the single-slope approach, and this effect is significantly lower for the self-calibrating approach. Larger error values in Figure 5b compared to Figure 5a can be explained by a smaller level of the DRS signals detected in vivo owing to higher extinction.

### 3.3. Reconstruction of Skin Chromophores and Scattering Properties

**Figure 6:**
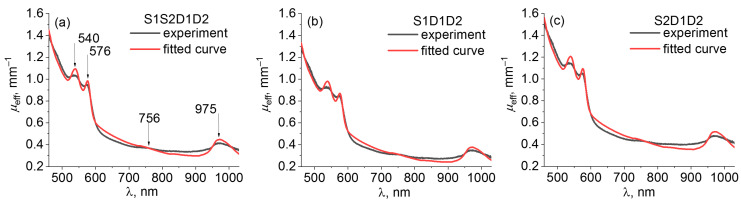
Fitting the extinction spectra of the in vivo human palm obtained from unperturbed measurements using (**a**) S1S2D1D2, (**b**) S1D1D2, and (**c**) S2D1D2 approaches with the reconstructed extinction spectra using Equations (11)–(13).

Examples of fitting the extinction spectra of the in vivo human palm calculated using the self-calibrating and single-slope approaches for unperturbed DRS data with expressions given by Equations (1), (2), and (10) using Equations (11)–(13) are shown in Figure 6. The fitting curve tracks the most pronounced visible features of the extinction spectrum: oxyhemoglobin peaks in the visible spectral range at 540 and 576 nm, deoxyhemoglobin peak at 756 nm, water absorption peak at 975 nm, and an overall decrease in the extinction coefficient from short to long wavelengths due to a decrease in absorption and scattering. However, there is some discrepancy between the reconstructed and experimentally obtained extinction spectra caused by the significant simplification of the applied model of light transfer in a human palm.

**Figure 7:**
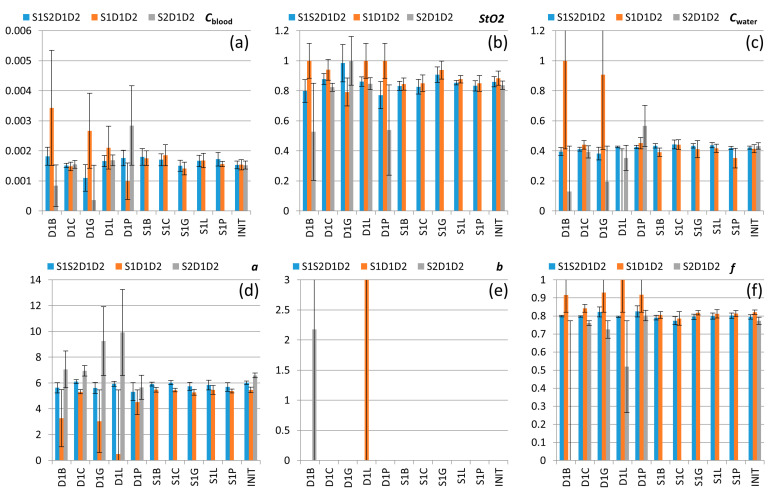
Values of $C_{blood}$ (**a**), $StO_{2}$ (**b**), $C_{water}$ (**c**), *a* in mm^−1^ (**d**), b (**e**), and f (**f**) reconstructed from the experimental extinction spectra using S1S2D1D2, S1D1D2, and S2D1D2 approaches and averaged over 3 measurements for each of the 10 perturbations (Table 1) and over 6 measurements for INIT data. Error bars show deviations in the corresponding reconstructed values in the series of the experiment. All values of b are below 10^−8^, except the two corresponding to the perturbations D1B and D1L, which lead to largest deviations in the reconstructed extinction spectra from the unperturbed one (see Figure 4c,e).

Figure 7 shows the values of blood $C_{blood}$ and water $C_{water}$ content, tissue oxygenation $StO_{2}$, and scattering properties a, *b*, and *f* of human palm reconstructed from fitting the experimental extinction spectra which were obtained using different measurement approaches (self-calibrating S1S2D1D2 and single-slope S1D1D2, S2D1D2) from DRS data measured under different instrumental perturbations listed in Table 1. The reconstructed values obtained for unperturbed (INIT) data are in agreement with typical skin physiological parameters [18,43]: $StO_{2}$ is about 0.8 and $C_{blood}$ and $C_{water}$ are around 0.002 and 0.4, respectively. The reconstructed values of *a*, *b*, and *f* yield the $\mu_{s}^{\prime }\left(\lambda \right)$ dependence, which is in agreement with the reduced scattering spectra reported in [43]: the short wave range of the reconstructed spectrum tends to the typical values reported for epidermis owing to a smaller probing depth in this range, while in the NIR range the recovered spectrum corresponds well to the $\mu_{s}^{\prime }$ spectrum reported for dermis.

**Figure 8:**
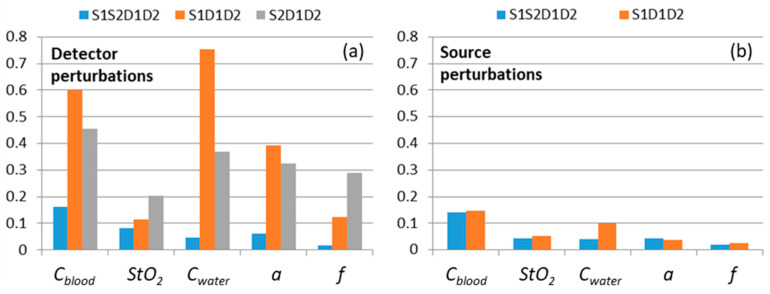
Relative deviations in $C_{blood}$, $StO_{2}$, $C_{water}\;,a$, and f values reconstructed using self-calibrating (S1S2D1D2) and single-slope (S1D1D2 and S2D1D2) approaches from the unperturbed measurement values. Relative deviations are averaged over different types of detector (**a**) and source (**b**) perturbations for each reconstructed value. Note that the plot (**b**) does not contain S2D1D2 data since perturbations introduced to the S1 channel provide no impact on these measurements.

As follows from the analysis of extinction coefficient deviations, skin optical parameters reconstructed from S1S2D1D2 extinction spectra demonstrate high stability for all DRS data obtained under all possible perturbations in which the deviation does not exceed 16%. In contrast, the parameters reconstructed from the single-slope data S1D1D2 and S2D1D2 demonstrate stability only for source perturbations and unperturbed data. These results are summarized in Figure 8, showing relative deviations in different skin characteristics obtained with all source (S1L, S1C, S1B, S1G, S1P) and all detector (D1L, D1C, D1B, D1G, D1P) perturbations. These plots demonstrate low average variations (less than 16%) of the parameters reconstructed from self-calibrating data for all types of perturbations, while detector perturbations may result in high variations (up to several times for particular perturbations) of reconstructed values from single-slope data (Figure 8a).

## 4. Discussion

In this study we compared the capabilities of single- and dual-slope approaches in DRS to resist different perturbations that may occur during measurements. An experimental setup for wide-band DRS with a fiber-optic contact probe capable of employing a self-calibrating approach was constructed. This system contains a broadband fiber-optic source allowing for diffuse reflectance measurements in a wide VIS-NIR band (460–1030 nm). The upper wavelength range boundary is limited by the detector sensitivity curve, while detection in the short wavelength range is limited by strong probing light attenuation in biotissue. The self-calibrating scheme is based on symmetrical source-detector measurements performed through two fiber-optic switches for two source and two detection fibers of the probe (Figure 1). In order to fit the spectrometer dynamic range for the whole wavelength range for short (2 mm) and long (4 mm) SDDs, we applied different exposure times of 15 and 80 ms, respectively.

Different instrumental perturbations have been introduced into source and detector channels including attenuation, fiber bending, and corrupting probe–tissue interface in order to compare resistance to them of self-calibrating and single-slope approaches in phantom and in vivo studies. Both approaches have been applied to analyze the corresponding extinction spectrum deviations originating from the applied perturbation during DRS measurements. The results of phantom and in vivo studies have shown (Figure 3 and Figure 4) that both approaches have resistance to instrumental perturbations introduced into the source channel (S1L, S1C, S1B, S1G, S1P). At the same time, perturbations introduced into the detection channel (D1L, D1C, D1B, D1G, D1P) may lead to significant deviations in the extinction spectra calculated by the single-slope approach (S1D1D2 or S2D1D2), while the self-calibrating approach (S1S2D1D2) demonstrated much higher resistance. This can be explained by the fact that Equation (5) for single slopes contains the ratio of detector transient characteristics and excludes source transient characteristics. However, Equations (6) and (7) are written under the assumption that the transient characteristics of both detectors are equal, therefore, perturbations introduced into one of the detector channels lead to the corruption of the evaluated extinction spectra. In contrast to this, in Equation (9) for the extinction spectrum calculated using self-calibrating approach, the transient functions for both sources and detectors are reduced. Figure 5a also demonstrates higher variations of extinction spectra calculated by the single-slope approach in comparison with the self-calibrating approach even for unperturbed data and all source perturbations. This effect is explained by residual instrumental perturbations (residual fiber bending, variations in SMA-connectors, etc.) that remained after perturbations introduced into source and detector channels during phantom studies. Figure 3f and Figure 4f also demonstrate the imperfections in the detection channels of the designed DRS system seen by the discrepancies in the opposite sign between the values of $\mu_{eff}$ reconstructed from the unperturbed spectra in self-calibrating mode versus those for single-slope configurations S1D1D2 and S2D1D2.

Reconstruction of the biotissue properties from the obtained extinction coefficient spectra demonstrated that the self-calibrating approach provides reliable values with average deviations not exceeding 16% for all the considered perturbations. In this connection, if the spectral changes induced by instrumental perturbations in clinical conditions are similar to those we employed in this study, one can expect approximately the same accuracy of the self-calibrating technique. However, it should be noted that the DRS measurements in subcutaneous tissues are sensitive to the applied pressure of a DRS probe. Excessive pressure of a DRS probe may result in a significant change in tissue optical properties, while a loose probe–tissue contact may lead to a slight probe shift during the measurement procedure. Both effects may result in errors in the reconstructed physiological parameters. To avoid these effects, in the designed system, the DRS probe was equipped with a pressure control unit allowing it to keep the optimal pressure during measurements.

In the present study we applied the simplest reconstruction technique of skin optical properties based on a standard MATLAB minimization function, and the model of DRS spectra was taken from the diffusion approximation of radiation transfer theory for infinite and homogeneously scattering and absorbing medium. This model was applied to assess the instability of reconstructed tissue property values caused by instrumental variations; however, the reconstructed *µ_eff_* spectrum has noticeable discrepancy with the experimental extinction spectrum obtained from the unperturbed measurements (Figure 6). We also limited the number of chromophores that contribute to the absorption spectrum in the described wavelength range and did not consider lipids because we focused on characterizing the chromophores of human palm, where the content of lipids is typically moderate and located mainly in hypodermis. Since our DRS system has SDDs of units in millimeters, it is mostly sensitive to superficial chromophores of skin such as water, blood, and melanin and to a lesser extent, to lipids in hypodermis at depths exceeding 1–2 mm. For a more precise reconstruction, the presence of the biotissue boundary and the skin layered structure should be taken into account, and more sophisticated algorithms of reconstruction should be used. It is essential for broadband DRS measurements in which light in the VIS and NIR spectral ranges penetrates to different depths in tissue. Monte-Carlo modeling of light transport can be used to take into account layered skin structure [44,45], and tissue properties can be derived using machine learning based on advanced theoretical and numerical models of light transport [46,47,48,49].

## 5. Conclusions

A comparative analysis of the sensitivity of single- and dual-slope (self-calibrating) approaches in DRS was performed using a custom-built wideband 460–1030 nm DRS setup in phantom and in vivo studies. Different instrumental perturbations have been introduced into source and detector channels in order to compare the stability of self-calibrating and single-slope approaches toward uncontrolled attenuations in individual channels, optical fiber bending, and optical inhomogeneities at the probe–tissue interface. Both single-slope and self-calibrating approaches have demonstrated high stability to perturbations introduced into the source channels. Perturbation in the detection channels may lead to significant deviations in the extinction spectra recovered from the measured back-reflectance spectra by the single-slope approach, however, the self-calibrating approach has demonstrated high stability for all types of perturbations. Reconstruction of the biotissue properties from the obtained extinction coefficient spectra demonstrated that the self-calibrating approach provides reliable values with average deviations not exceeding 16% for all the considered perturbations. Thus, we can conclude that the self-calibrating approach can be applied to DRS to provide robust measurements insensitive to instrumental perturbations in a wide VIS-NIR spectral band.

## Figures and Tables

**Table 1:** Description of instrumental perturbations used in phantom and in vivo experiments.

Perturbation Index *P*	Description of Perturbation
D1L	Simulation of losses in detection channel. Ø105 µm fiber was inserted between detection fiber D1 and Switch 2.
D1C	Simulation of curving detection fiber. Detection fiber D1 is curved into a 50 mm radius ring.
D1B	Simulation of probe–tissue interface inhomogeneity in detection channel. A blue sticker was inserted between detection fiber D1 end and investigated object
D1G	Simulation of probe–tissue interface inhomogeneity in detection channel. A green sticker was inserted between detection fiber D1 end and investigated object
D1P	Simulation of probe–tissue interface inhomogeneity in detection channel. A pink sticker was inserted between detection fiber D1 end and investigated object
S1L	Simulation of losses in source channel. Ø105 µm fiber was inserted between source fiber S1 and Switch 1.
S1C	Simulation of bending source fiber. Source fiber S1 is curved into 50 mm radius ring.
S1B	Simulation of probe–tissue interface inhomogeneity in source channel. A blue sticker was inserted between source fiber S1 end and investigated object
S1G	Simulation of probe–tissue interface inhomogeneity in source channel. A green sticker was inserted between source fiber S1 end and investigated object
S1P	Simulation of probe–tissue interface inhomogeneity in source channel. A pink sticker was inserted between source fiber S1 end and investigated object
INIT	No perturbations were implemented to fibers

## Data Availability

The data presented in this study are available on a reasonable request from the corresponding author.
